# Soy-RepViT: a lightweight deep learning framework for fine-grained recognition of soybean growth habits

**DOI:** 10.3389/fpls.2026.1954156

**Published:** 2026-09-30

**Authors:** Dongbo Liu, Daohan Cui, Hang Liu, Tianyuan Song, Zhanguo Zhang, Qingshan Chen, Fanchuang Li, Rongsheng Zhu

**Affiliations:** 1 College of Intelligence Science and Engineering, Northeast Agricultural University, Harbin, Heilongjiang, China; 2 State Key Laboratory of Smart Farm Technologies and Systems, Harbin, China; 3 College of Agriculture, Northeast Agricultural University, Harbin, Heilongjiang, China; 4 School of Resources and Environment, Northeast Agricultural University, Harbin, Heilongjiang, China

**Keywords:** fine-grained classification, hierarchical classification, lightweight deep learning, plant phenotyping, soybean growth habit

## Abstract

Soybean growth habit is generally classified into determinate, indeterminate, and semi-determinate types, and its accurate recognition is important for breeding selection, high-density cultivation, and agronomic management. However, the semi-determinate type is intermediate between determinate and indeterminate types and shows continuous transitional characteristics with the indeterminate type in stem elongation, upper internode length, and pod distribution, making fine-grained discrimination between these two types difficult. Practical deployment also requires lightweight, efficient models. This study developed a lightweight fine-grained recognition framework for soybean growth habit from standardized plant images. The framework used RepViT-M1 as an efficient feature extraction backbone and integrated a multi-scale efficient channel attention module (MS-ECA) to enhance discriminative channel features, a Fine-grained Disambiguation Adapter (FDA) to recalibrate non-determinate features using class prototypes and reduce indeterminate/semi-determinate confusion, and a hierarchical classification head to incorporate the agronomic hierarchy between determinate and non-determinate types. The dataset comprised 944 original soybean plant images and was expanded to 4,709 images through offline augmentation. The model was evaluated using five-fold cross-validation and an independent test set. The proposed model achieved 97.45% accuracy on the independent test set, improving the RepViT-M1 baseline by approximately 3 percentage points. Accuracy for the indeterminate type increased from 91.30% to 97.83%, indicating that the model effectively reduced confusion between indeterminate and semi-determinate types. The model had 4.94 M parameters, required 0.82 GFLOPs, and reached 108.75 FPS, demonstrating a favorable balance between accuracy and efficiency. The proposed framework improves fine-grained soybean growth habit recognition at low computational cost and demonstrates potential for standardized, high-throughput phenotypic identification through on-device Android inference.

## 1 Introduction

Soybean is an important oilseed and protein crop, and its plant architecture is closely associated with yield formation and agronomic adaptability (Li et al., 2024). Stem growth habit is a key trait determining plant architecture and is generally classified into determinate, indeterminate, and semi-determinate types (Xiong et al., 2023; Liu et al., 2010). In determinate plants, the main stem ceases elongation after forming a terminal inflorescence; in indeterminate plants, the main stem continues to grow after flowering; whereas semi-determinate plants exhibit characteristics intermediate between the two (Bernard, 1972). Given its close association with key agronomic traits—such as plant height, main stem node number, and maturity progression (Heatherly and Smith, 2004) — accurate identification of soybean growth habit is of great importance for germplasm evaluation, breeding selection, and agronomic management.

Traditional growth habit identification primarily relies on the morphology of the main stem apex and stem elongation characteristics, and is typically performed through manual observation. Although this approach is intuitive, it is inefficient for large-scale surveys and heavily dependent on the experience of the evaluator (Araus and Cairns, 2014). In particular, indeterminate and semi-determinate types exhibit continuous transitional characteristics in main stem elongation status, upper internode length, and pod distribution patterns. As a result, some boundary samples show a high degree of phenotypic similarity, leading to inconsistencies in manual identification. Therefore, there is an urgent need to develop an efficient, rapid, and accurate method for soybean growth habit identification.

In recent years, deep learning has become a major technological approach for plant image analysis and phenotypic recognition (Ubbens and Stavness, 2017). Classical convolutional neural networks (CNNs), such as AlexNet (Krizhevsky et al., 2012), VGG (Simonyan and Zisserman, 2015), and ResNet (He et al., 2016), have laid the foundation for agricultural vision tasks, while recent review studies have systematically summarized the rapid advances of deep learning in image-based plant phenotyping (Murphy et al., 2024; Maraveas, 2024). These models have been widely applied to tasks such as plant disease recognition, plant organ detection, growth stage classification, and seed quality inspection. For example, Mohanty et al. employed AlexNet and GoogLeNet to identify leaf disease images from the PlantVillage dataset (Mohanty et al., 2016), whereas Younis et al. utilized Faster R-CNN to detect and annotate multiple categories of plant organs in digitized herbarium specimens (Younis et al., 2020). Qin et al. conducted a rice phenological stage recognition study using ResNet-50 as the backbone network (Qin et al., 2023), while Wang et al. developed a dual-path convolutional neural network, Corn-seed-Net, by integrating VGG16 and ResNet50 for corn seed defect detection (Wang et al., 2022). These studies demonstrate that deep learning methods are well suited to agricultural image classification and phenotypic recognition tasks. With the growing demand for edge devices and field-deployable applications, lightweight convolutional networks have increasingly emerged as an important research direction in agricultural vision. Representative lightweight CNNs, such as ShuffleNet (Zhang et al., 2018), EfficientNet (Tan and Le, 2019), and MobileNetV3 (Howard et al., 2019), reduce parameter counts and computational costs through efficient architectural designs while maintaining competitive recognition performance. In agricultural applications, Zhao et al. developed a deep learning-based real-time recognition system for full-surface soybean seed defects and enabled real-time inference on edge devices (Zhao et al., 2021); Liu et al. utilized MobileNetV3 for cucumber leaf disease recognition in natural field scenes, further demonstrating the application potential of lightweight networks under complex backgrounds and in mobile deployments (Liu et al., 2023). Beyond convolutional neural networks, Vision Transformers have also attracted increasing attention in image recognition in recent years. ViT leverages self-attention mechanisms to model global image information and offers advantages in capturing complex structural relationships and long-range dependencies (Dosovitskiy et al., 2021). In agricultural image analysis, Borhani et al. proposed a Vision Transformer-based method for automated plant disease classification (Borhani et al., 2022), while Bi et al. further applied Transformers to early soybean yield prediction using time-series images (Bi et al., 2023). However, pure Transformer models typically require large-scale datasets and substantial computational resources, which limits their direct deployment in resource-constrained settings. To balance model accuracy and runtime efficiency, Wang et al. proposed RepViT, re-examining the structural design of mobile CNNs from a ViT perspective while maintaining strong feature representation at low latency (Wang et al., 2024). In phenotypic analysis, Wang et al. (2025) embedded RepViT Blocks into the YOLO11-seg segmentation framework for non-contact measurement of sunflower disk inclination, achieving high measurement accuracy with reduced parameter count and computational cost.

Overall, although deep learning applications in agriculture have progressed rapidly, existing work remains largely focused on plant disease recognition, seed detection, and the automated extraction of conventional phenotypic traits such as plant height and pod number (Chen et al., 2023; Meraj et al., 2024). In soybean research, Zhou et al. proposed SPP-extractor based on an improved YOLOv5s for phenotypic extraction of dense, overlapping plants at maturity (Zhou et al., 2023), while Zhang et al. utilized YOLOv8-series models to automatically measure phenotypic traits such as pods, stems, and branches (Zhang et al., 2024). However, the above studies primarily target quantifiable morphological traits, and research remains limited for growth habit—a fine-grained recognition task characterized by subtle phenotypic differences and continuous transitions across class boundaries (Sun et al., 2025). Particularly, indeterminate and semi-determinate growth habits exhibit highly similar phenotypes, and conventional convolutional networks are prone to feature aliasing in feature space, making it difficult to establish stable fine-grained discriminative boundaries. Therefore, developing a soybean growth habit classification model that combines fine-grained discriminative capability with lightweight deployability remains of significant importance. To address these challenges, this study developed a lightweight fine-grained classification framework for soybean growth habit recognition. The framework uses RepViT-M1 as an efficient feature extraction backbone and integrates MS-ECA, FDA, and a hierarchical classification head to enhance key discriminative features, alleviate boundary confusion between similar growth habit types, and incorporate the agronomic hierarchy of soybean growth habits. The main contributions of this work are as follows:

1. A lightweight fine-grained classification model was developed for soybean growth habit classification by integrating multi-scale efficient channel attention (MS-ECA), a Fine-grained Disambiguation Adapter (FDA), and a hierarchical classification head to refine the recognition process through feature enhancement, fine-grained discrimination, and hierarchical discrimination. As a result, the proposed model improves the discrimination of ambiguous boundary samples while maintaining low computational complexity;
2. A standardized soybean growth habit image dataset was established from 944 original plant images covering determinate, indeterminate, and semi-determinate growth habits and was expanded to 4,709 images through offline augmentation. The original images were acquired using the standardized imaging platform under controlled conditions. Five-fold cross-validation and an independent test set were constructed at the plant level to ensure the reliability and reproducibility of the experimental results;
3. Comprehensive performance evaluation and on-device deployment validation were conducted. The proposed model achieved an accuracy of 97.45% on the independent test set while maintaining 4.94 M parameters, 0.82 GFLOPs, and an inference speed of 108.75 FPS, demonstrating an effective balance between classification accuracy and deployment efficiency. Furthermore, an Android application using an ONNX model was deployed on a smartphone to demonstrate the practical applicability of the proposed framework in standardized high-throughput identification scenarios.

## 2 Materials and methods

The experimental workflow was organized sequentially from data processing through model construction, training, and deployment, with the overall workflow illustrated in **Figure 1**. **Figure 1A** covers standardized image acquisition, three-class annotation, preprocessing, and offline augmentation; **Figure 1B** presents the construction of the soybean growth habit classification network; **Figure C** describes data partitioning, training configuration, and the evaluation pipeline; **Figure 1D** includes comparison of attention mechanisms and Grad-CAM analysis; and Figure 1E demonstrates the Android-based on-device inference application.

**Figure 1:**
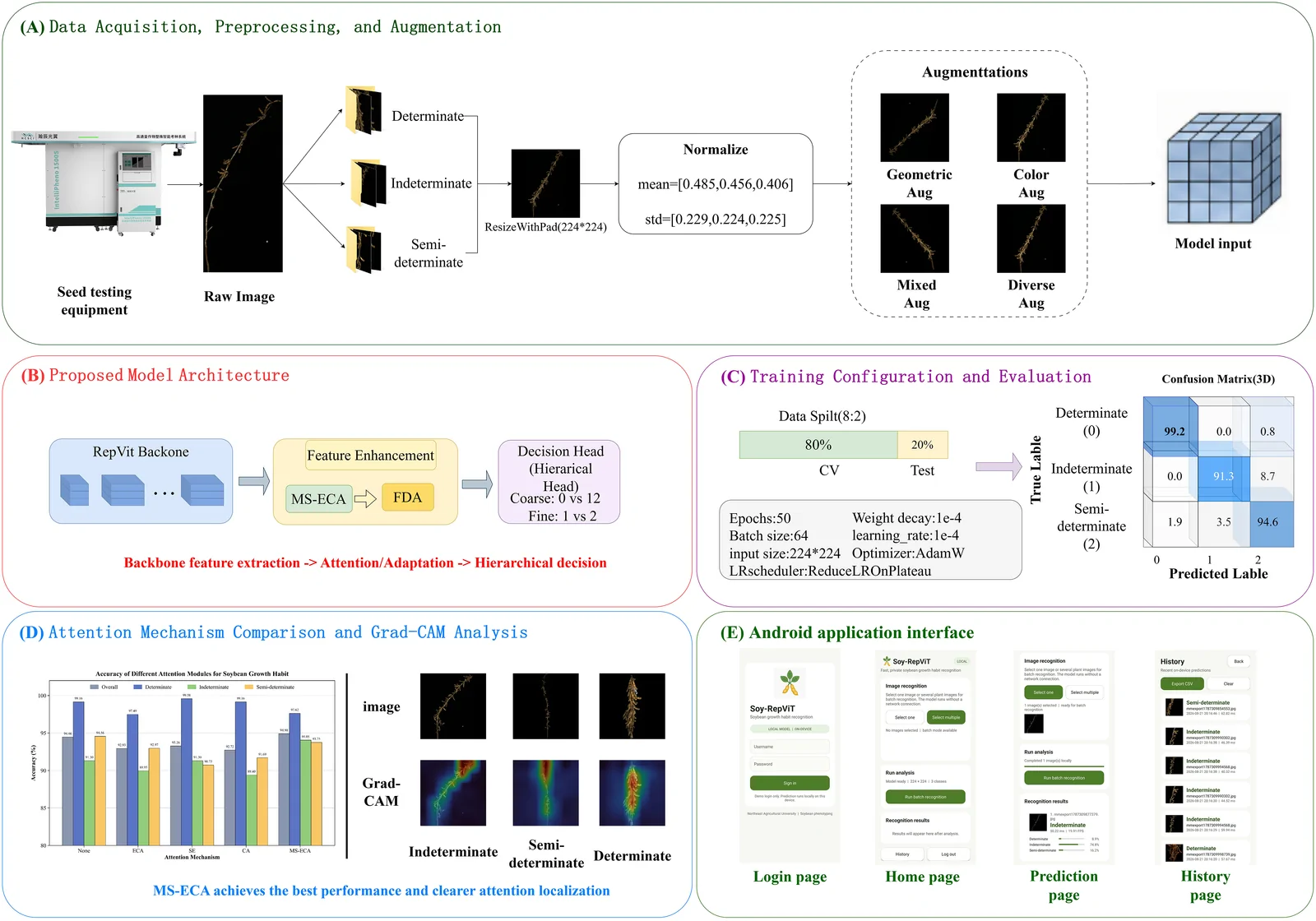
Overall workflow of the soybean growth habit classification framework. **(A)** Data acquisition and preprocessing. **(B)** Model construction. **(C)** Training and evaluation. **(D)** Model analysis. **(E)** Android on-device inference application.

### 2.1 Dataset acquisition and annotation

As shown in **Figure 1**, the soybean plant samples used in this study were collected from mature plants at Xiangyang Farm, Northeast Agricultural University (Harbin). The image dataset was acquired using a seed testing instrument, which was used as a standardized imaging platform with controlled illumination and a fixed imaging setup. A total of 944 samples were obtained, covering three growth habits: determinate, indeterminate, and semi-determinate. For each plant, a high-resolution color image was captured from the primary viewing angle. Because the original images varied in resolution, all images were uniformly resized during preprocessing.

All annotations were performed independently by agronomic experts with expertise in soybean breeding. Each sample was classified primarily based on main stem apical growth status, pod distribution patterns, and internode elongation.

As shown in **Figure 2**, representative images illustrate the three growth habits. Determinate plants stop elongating after a terminal inflorescence develops at the main stem apex, with relatively short internodes. Pods are predominantly concentrated in the middle and upper main stem, and a conspicuous pod cluster often forms at the top (**Figure 2A**). Indeterminate plants continue to elongate for several weeks after flowering, with relatively long internodes. Pods become progressively sparser from bottom to top, and no conspicuous pod cluster typically forms at the apex (**Figure 2B**). Semi-determinate plants exhibit intermediate characteristics: the main stem apex continues to extend to some extent after flowering, but to a markedly lesser degree than in indeterminate plants. Internodes at the top are relatively thick and short, pods are relatively concentrated, and transitional pod distribution patterns are observed (**Figure 2C**). These typical phenotypic differences provide an important basis for manual annotation and constitute the phenotypic foundation for fine-grained recognition in this study.

**Figure 2:**
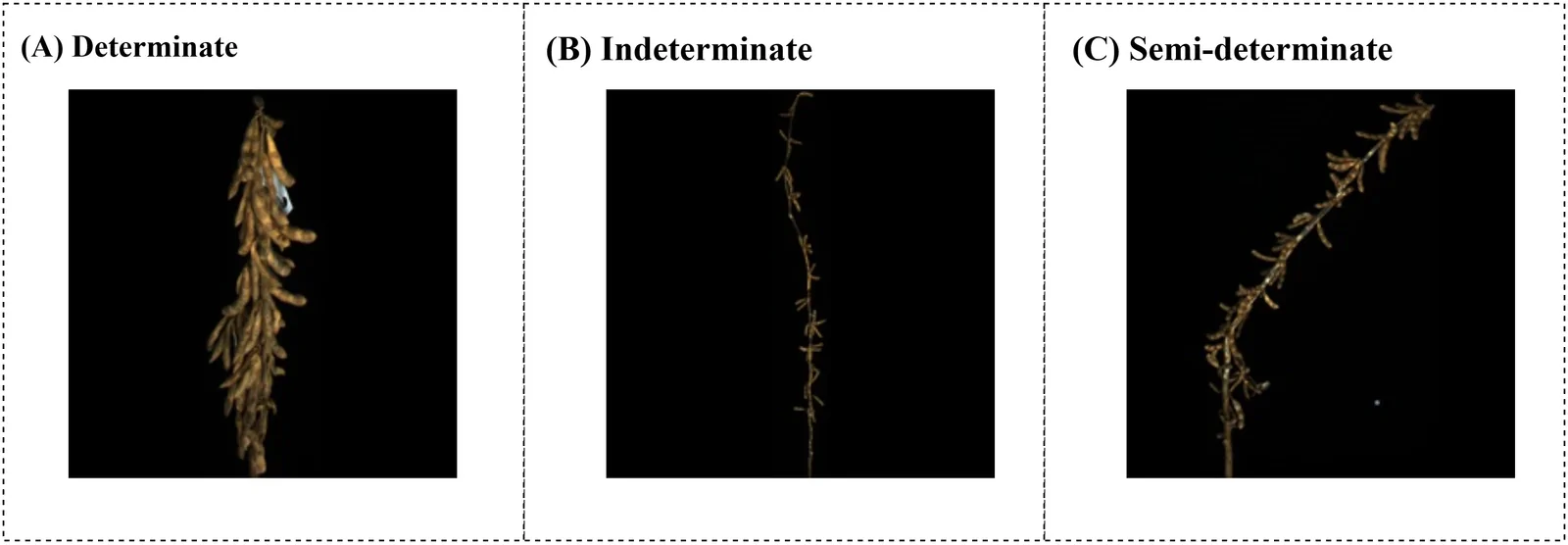
Representative examples of three soybean growth habits. **(A)** Determinate. **(B)** Indeterminate. **(C)** Semi-determinate.

As shown in **Figures 1A** and **3A**, the preprocessing stage consisted of two steps. First, each original image underwent aspect-ratio-preserving scaling and padding: the shorter side was resized to 224 pixels, the longer side was scaled proportionally, and black pixels were padded on all sides to obtain a 224 × 224 square image. Normalization was then applied to maintain consistency with the pretrained weights (Deng et al., 2009). To improve result stability and reduce the computational overhead of online augmentation, offline data augmentation was adopted in this study. Before dataset partitioning, the normalized original images were augmented (**Figure 1A**). The augmentation strategies included random rotation, horizontal flipping, brightness/contrast adjustment, Gaussian blur, and Gaussian noise (Yang et al., 2023; Zeng, 2024). Several augmented images were generated from each original image, all retaining the original plant ID.

Data partitioning was performed at the plant-ID level. Original and augmented images associated with the same plant ID were assigned to the same subset to prevent data leakage. Specifically, 20% of the 944 plants were randomly selected as the independent test set, and the remaining 80% were used for five-fold cross-validation, with an approximate training-to-validation ratio of 8:2 in each fold. The validation set was used for model selection and hyperparameter determination, including the selection of the FDA parameters ρ and τ. After the model configuration was finalized, the baseline model, the complete model, and the pre-defined ablation variants were evaluated on the same independent test set, which was not used for model selection or hyperparameter tuning. The training set included all images assigned to the corresponding plant IDs, whereas the validation and test sets contained only the padded and normalized original images without augmentation. The numbers of original and augmented images for each class are summarized in **Table 1**.

**Table 1:** Statistics of image sample numbers by class.

Class	Code	Original images	Augmented images	Total images	Proportion (%)
Determinate	0	137	1175	1312	27.9%
Indeterminate	1	299	1399	1698	36.1%
Semi-determinate	2	508	1191	1699	36.0%
Total	—	944	3765	4709	100.0%

### 2.2 Model architecture

In this study, RepViT-M1 was used as the network backbone, and MS-ECA, FDA, and a hierarchical classification head were integrated to construct a hierarchical classification model for soybean growth habit. In the implemented architecture, RepViT-M1 extracted multi-scale features, MS-ECA enhanced responses of critical channels, FDA primarily mitigated fine-grained confusion between indeterminate and semi-determinate types, and the hierarchical classification head first separated determinate from non-determinate plants and then further discriminated indeterminate from semi-determinate types. The overall architecture and detailed components of the model are illustrated in **Figure 3**.

**Figure 3:**
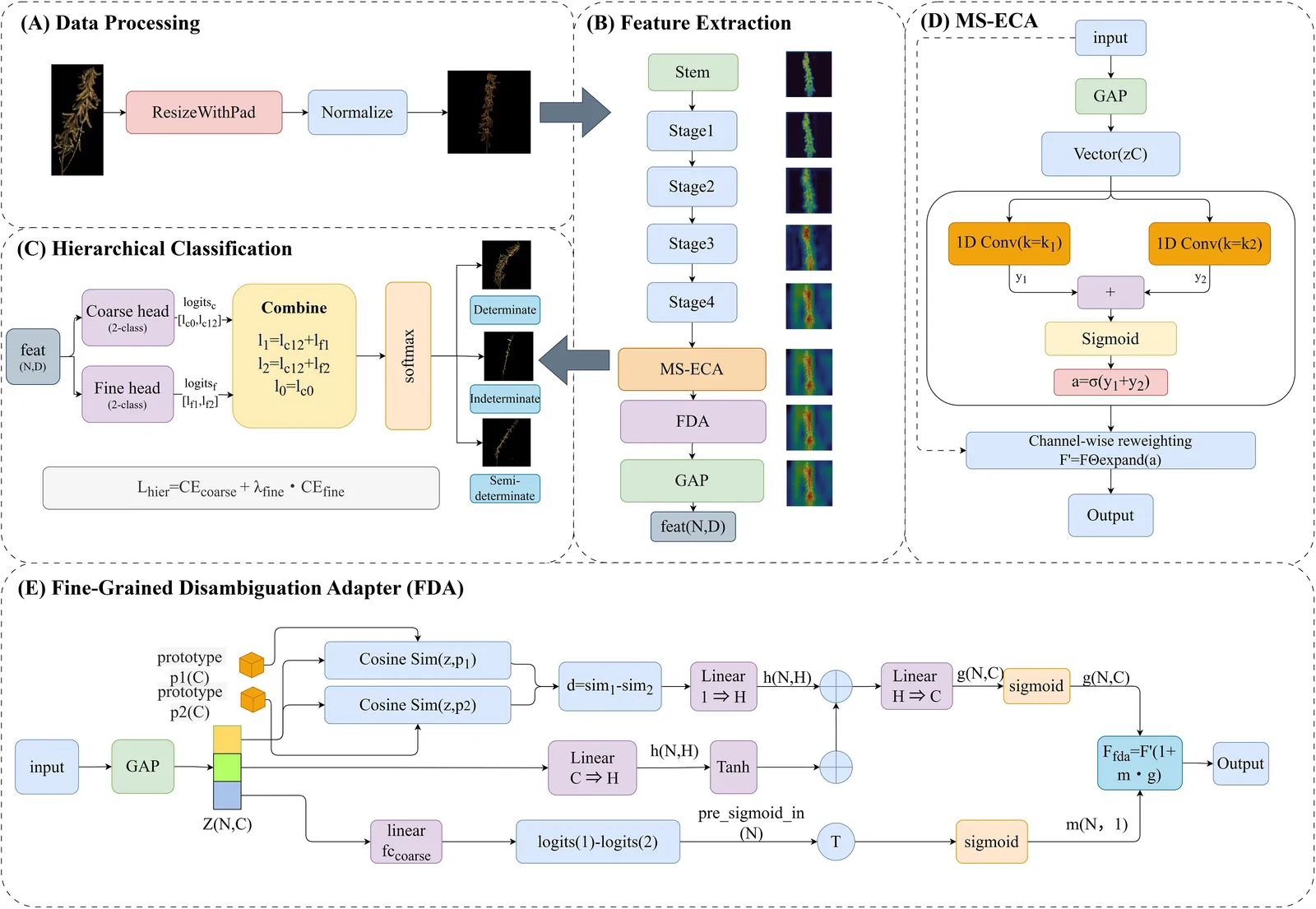
Overall architecture of the soybean growth habit classification model. **(A)** Input module. **(B)** RepViT feature extraction and enhancement module. **(C)** Hierarchical classification module. **(D)** MS-ECA module. **(E)** FDA module.

#### 2.2.1 RepViT-M1 backbone network

RepViT-M1 is a lightweight CNN–ViT hybrid backbone that reduces inference overhead while maintaining accuracy through structural re-parameterization (Wang et al., 2024). The overall structure is illustrated in **Figure 3B**. The Stem module comprises two cascaded 3×3 convolutions with a stride of 2, which rapidly downsample 224 × 224 inputs to 56 × 56. The network then passes through four feature extraction stages, where spatial resolution is halved stage by stage and the number of channels increases accordingly. Each stage contains RepViT Blocks that progressively expand the receptive field through stacking, enabling extraction of multi-scale features from local textures to global semantics.

#### 2.2.2 MS-ECA module

Standard ECA (Wang et al., 2020) employs only single-scale 1D convolution to capture channel dependencies, making it difficult to simultaneously accommodate the multi-scale requirements of discriminative regions across determinate, indeterminate, and semi-determinate classes. To address this limitation, a multi-scale efficient channel attention (MS-ECA) module was introduced (**Figure 3D**). Given a RepViT output feature map F ∈ ${\mathbb{R}}^{B\times C\times H\times W}$, MS-ECA first applies global average pooling to obtain a channel descriptor z ∈ ${\mathbb{R}}^{B\times C}$. z is then fed in parallel into two fast 1D convolutions with different kernel sizes to capture multi-scale local cross-channel interactions. The outputs of the two branches are summed and passed through a sigmoid activation to generate multi-scale channel attention weights a ∈ ${\mathbb{R}}^{B\times C}$. Finally, the original feature F is multiplied with a in a channel-wise manner to obtain the weighted feature map, as given in Equation 1:

(1) $$\left\{ \begin{array}{l} z=GAP(F),\\ a=\sigma (\text{Conv}1{\text{D}}_{k_{1}}(z)+\text{Conv}1{\text{D}}_{k_{2}}(z)),\\ F^{\prime }=F⊙a. \end{array}\right.$$

Where GAP denotes global average pooling; *z* is the channel descriptor obtained from the input feature map *F*; $k_{1}$ and $k_{2}$ denote the kernel sizes of the two parallel 1D convolutions; *σ* (·) denotes the sigmoid function; *a* denotes the multi-scale channel attention weights; ⊙ represents channel-wise multiplication; and $F^{\prime }$ denotes the recalibrated feature map.

#### 2.2.3 FDA module

Indeterminate and semi-determinate types exhibit continuous transitional characteristics in main stem extension, upper internode length, and pod distribution patterns, and share many similar phenotypic regions. As a result, the model tends to produce overlapping responses in the deep feature space, making it difficult to establish stable fine-grained discriminative boundaries. To enhance modeling of intra-class differences within non-determinate types, a Fine-grained Disambiguation Adapter (FDA) was introduced (**Figure 3E**). FDA takes the MS-ECA output feature map $F^{\prime }$ as input and applies global average pooling to obtain a channel descriptor z. Considering the continuous transitional relationship between indeterminate and semi-determinate types in agronomic phenotypes, FDA introduces two learnable class prototypes, $p_{1}$ and $p_{2}$, which represent the typical channel response patterns of indeterminate and semi-determinate types in the high-level feature space, respectively (Snell et al., 2017). Both prototypes were randomly initialized and jointly optimized with the network via backpropagation during training. The similarity difference d between z and the two prototypes is then computed to indicate which class prototype the current sample more closely resembles. On this basis, FDA generates a channel gating vector g and recalibrates the features using a coarse-grained gating factor m. Because the boundaries of the determinate class are relatively clear, FDA primarily targets intra-class differences within non-determinate types between indeterminate and semi-determinate plants, thereby enhancing critical channel responses related to boundary discrimination and improving fine-grained discriminative ability for boundary samples exhibiting continuous transitional characteristics. This process is formulated as Equation 2:

(2) $$\left\{ \begin{array}{l} d\&=\frac{z\cdot p_{1}}{∥z∥∥p_{1}∥}-\frac{z\cdot p_{2}}{∥z∥∥p_{2}∥},\\ h=tanh(W_{z}z+W_{d}d+b),\\ g=\sigma (W_{g}h),\\ m\&=\sigma (\frac{{\left(f_{coarse}(z)\right)}_{1}-{\left(f_{coarse}(z)\right)}_{0}}{\tau }),\\ F_{fda}=F^{\prime }⊙(1+mg). \end{array}\right.$$

Where *h* is the intermediate hidden representation generated from *z* and *d*; $W_{z}$, $W_{d}$, and $W_{g}$ are learnable weight parameters; *b* is a bias term; *τ* is a temperature coefficient; *σ* (·) denotes the sigmoid function; ⊙ denotes element-wise multiplication; and ${\left(f_{coarse}(z)\right)}_{1}$ and ${\left(f_{coarse}(z)\right)}_{0}$ denote the response values of the determinate and non-determinate classes output by the coarse-grained classification head, respectively.

#### 2.2.4 Hierarchical classification head and hierarchical loss function

Leveraging the natural hierarchical distinction between the determinate class and the other two growth habit classes in soybean, a hierarchical classification head was designed (**Figure 3C**), consisting of coarse-grained and fine-grained branches (Silla and Freitas, 2011; Yan et al., 2015). Global average pooling is applied to the FDA output feature map $F_{fda}$ to obtain a feature vector f ∈ ${\mathbb{R}}^{B\times C}$, which is fed into both branches simultaneously. The coarse-grained head employs a two-layer MLP with Dropout, BatchNorm, and ReLU to produce 2-dimensional logits corresponding to the determinate and non-determinate classes, whereas the fine-grained head uses an MLP of the same structure to produce 2-dimensional logits corresponding to the indeterminate and semi-determinate classes. The final prediction logits are combined as Equation 3:

(3) $$l_{0}=l_{c0},\;l_{1}=l_{c12}+l_{f1},\;l_{2}=l_{c12}+l_{f2}.$$

Where $l_{0}$, $l_{1}$, and $l_{2}$ denote the final logits of the three growth habit classes; $l_{c0}$ and $l_{c12}$ denote the coarse-grained logits, and $l_{f1}$ and $l_{f2}$ denote the fine-grained logits.

The model was optimized using a hierarchical loss function, as formulated in Equation 4:

(4) $$L=CE(l_{c},y_{coarse})+1_{\left\{y>0\right\}}\cdot CE(l_{f},y_{fine}).$$

Where CE denotes the cross-entropy loss. In the first term, 1{y>0} represents the coarse-grained label, where y = 0 is mapped to 0 and y ∈ {1, 2} is mapped to 1. In the second term, 1{y>0} serves as an indicator function such that the fine-grained branch loss is accumulated only when y > 0. When y ∈ {1, 2}, y - 1 maps classes 1 and 2 to fine-grained labels 0 and 1, respectively. This design requires the model to first determine whether a plant belongs to the non-determinate category and then discriminate between indeterminate and semi-determinate types within non-determinate plants, consistent with the hierarchical decision logic of plant phenotyping experts. FDA does not introduce an additional supervised loss term, and its parameters are updated through backpropagation from L. At inference, the predicted class is obtained by taking the argmax of $l_{final}$.

### 2.3 Experimental setup

All experiments were conducted on a single NVIDIA GeForce RTX 3090 GPU with 24 GB of memory, running Windows 10, PyTorch 2.0, CUDA 11.8, and cuDNN 8.7. For model training, the data were split into training and independent test subsets at a ratio of 8:2, and five-fold cross-validation was performed on the training subset (Kohavi, 1995). The main training hyperparameters are listed in **Table 2**: the optimizer was AdamW, with both the initial learning rate and weight decay set to $1\times 10^{-4}$; the learning rate scheduler was ReduceLROnPlateau; training was conducted for 50 epochs with early stopping, automatic mixed precision (AMP), and gradient clipping. The batch size was set to 64 during training, whereas all inference-speed measurements were conducted with a batch size of 1. The main experiments used a fixed random seed of 6666 to ensure reproducible data partitioning, parameter initialization, and training comparisons. To assess robustness to random initialization, the baseline and complete models were additionally trained using multiple random seeds. The corresponding results are reported as mean ± standard deviation in **Supplementary Table S3**.

**Table 2:** Training hyperparameter configuration.

Hyperparameter	Value
Optimizer	AdamW
Initial learning rate	$1\times 10^{-4}$​
Weight decay	$1\times 10^{-4}$​
Training batch size	64
Number of epochs	50
Learning rate scheduler	ReduceLROnPlateau
Random seed	6666

### 2.4 Evaluation metrics

Classification performance was evaluated using accuracy, precision, recall, and F1-score (Rainio et al., 2024; Miller et al., 2024). The definitions of these metrics are given in Equations 5–8:

Accuracy measures the proportion of correctly classified samples to the total number of samples:

(5) $$\text{Accuracy}=\frac{TP+TN}{TP+TN+FP+FN}.$$

Precision measures the proportion of actually positive samples among all samples predicted as positive:

(6) $$\text{Precision}=\frac{TP}{TP+FP}.$$

Recall measures the proportion of correctly predicted samples among all actually positive samples:

(7) $$\text{Recall}=\frac{TP}{TP+FN}.$$

The F1-score is the harmonic mean of precision and recall, providing a comprehensive evaluation of classification performance, especially when class distributions are imbalanced (Chen et al., 2024):

(8) $$F1=\frac{2\cdot \text{Precision}\cdot \text{Recall}}{\text{Precision}+\text{Recall}}.$$

Where TP, FP, TN, and FN denote the numbers of true positives, false positives, true negatives, and false negatives, respectively. In addition, model complexity and efficiency were reported in terms of parameter count (Params, in M), computational cost (FLOPs, in G, at an input resolution of 224 × 224), and inference speed (FPS, with batch size = 1). Confusion matrices were presented as row-normalized percentages (Opitz, 2024).

## 3 Results

### 3.1 Model comparison results

To comprehensively evaluate the proposed Soy-RepViT model, it was compared with five mainstream lightweight networks: FastViT-S12 (Vasu et al., 2023), MobileNetV3-Large, EfficientNet-B0, ShuffleNetV2 ×1.0, and the plain RepViT-M1 baseline. In addition, two representative comparison methods were included: a prototype-based metric-learning method, ProtoNet (Snell et al., 2017), implemented with a ResNet-18 backbone, and the fine-grained recognition method DCL (Chen et al., 2019), implemented with a ResNet-50 backbone. All models were initialized with ImageNet-pretrained weights, used an input size of 224 × 224 pixels, and were trained under the same experimental configuration. To ensure a fair comparison, all models were evaluated using the same plant-level data partition, independent test set, and evaluation protocol. The original task-specific structures of ProtoNet and DCL were retained for comparison.

**Table 3** summarizes the overall accuracy, macro-averaged precision, recall, F1-score, parameter count, FP32 weight size, computational cost, and inference speed of all models. On the independent test set, Soy-RepViT achieved an accuracy of 97.45%, outperforming all comparison models. The plain RepViT-M1 baseline without the proposed modules achieved an accuracy of 94.46%, whereas the other lightweight networks mainly achieved accuracies between 92% and 93%. The two fine-grained comparison methods, ProtoNet and DCL, achieved accuracies of 87.09% and 90.79%, respectively, both lower than that of Soy-RepViT. Specifically, Soy-RepViT contained 4.94 M parameters, required 0.82 GFLOPs, and occupied approximately 19.80 MB in FP32 model weights. Compared with the RepViT-M1 baseline, the complete model increased the parameter count by only 0.22 M and the weight size by approximately 0.73 MB, while reducing GFLOPs from 0.85 to 0.82 and improving the inference speed from 104.74 to 108.75 FPS. These results indicate that Soy-RepViT effectively improves recognition accuracy while maintaining low computational and storage costs with only a modest increase in model size. Overall, the proposed model achieved a favorable balance between recognition performance and deployment efficiency.

**Table 3:** Performance comparison of different models on the independent test set.

Model	Acc (%)	Pre (%)	Rec (%)	F1 (%)	Params (M)	Weight size (MB, FP32)	GFLOPs	FPS
ProtoNet (ResNet-18)	87.09	87.80	87.87	87.74	11.24	45.05	3.63	1145.14
DCL (ResNet-50)	90.79	91.31	91.08	91.12	25.63	102.89	8.28	508.21
FastViT-S12	92.28	92.74	93.02	92.82	8.45	33.97	1.38	114.77
EfficientNet-B0	92.39	92.93	93.23	93.01	4.01	16.04	0.41	88.61
MobileNetV3-Large	93.37	93.62	93.79	93.70	4.21	16.84	0.23	231.17
ShuffleNetV2	92.07	92.59	92.80	92.67	1.26	5.04	0.15	230.66
RepViT-M1	94.46	94.69	95.01	94.80	4.72	19.07	0.85	104.74
Soy-RepViT	97.45	97.44	97.56	97.5	4.94	19.80	0.82	108.75

### 3.2 Ablation study results

To evaluate the individual contributions and complementary effects of MS-ECA (A), FDA (B), and the hierarchical classification head (C), eight pre-defined ablation configurations were designed under the same training setup. The module configurations were determined using the training and validation data, after which all ablation variants were evaluated on the same independent test set. The five-fold cross-validation results of the ablation experiments are provided in Supplementary Table S2, whereas the final results on the independent test set are reported in **Table 4**. The baseline RepViT-M1 achieved an overall accuracy of 94.46%, with an indeterminate-class accuracy of only 91.30%, indicating substantial confusion between indeterminate and semi-determinate types. When only a single module was added, MS-ECA, FDA, and the hierarchical classification head improved the overall accuracy to 94.90%, 95.75%, and 95.11%, respectively. Among them, FDA yielded the most pronounced improvement for the indeterminate class, reaching 97.29%, although the semi-determinate accuracy slightly decreased to 91.25%. The hierarchical classification head also improved the overall accuracy compared with the baseline model, indicating that incorporating the agronomic hierarchy benefited the classification process. Among the pairwise combinations, the MS-ECA + FDA configuration achieved the highest accuracy of 96.39%, suggesting that channel enhancement and class-prototype-guided feature recalibration provided complementary benefits. The complete model further improved the overall accuracy to 97.45%, with indeterminate and semi-determinate accuracies increasing to 97.83% and 95.62%, respectively. These results indicate that the three components address different aspects of the recognition task and achieve the best overall classification performance when used together.

**Table 4:** Ablation study results: overall and class-wise accuracies.

Model	MS-ECA (A)	FDA (B)	Hierarchical classification head (C)	Acc (%)	Determinate (%)	Indeterminate (%)	Semi-determinate (%)
1				94.46	99.16	91.30	94.56
2	✓			94.90	97.62	94.05	93.75
3		✓		95.75	99.20	97.29	91.25
4			✓	95.11	100.0	92.97	93.75
5	✓	✓		96.39	99.20	96.76	93.75
6	✓		✓	95.54	99.20	97.83	90.00
7		✓	✓	95.75	99.20	97.29	91.25
8	✓	✓	✓	97.45	99.20	97.83	95.62

### 3.3 Confusion matrix analysis

To analyze the misclassification distribution across classes and the improvement of the complete model over the baseline RepViT-M1, row-normalized percentage confusion matrices for both models on the independent test set are presented in **Figure 4**. For the baseline model, 8.7% of indeterminate samples were misclassified as semi-determinate, and 3.5% of semi-determinate samples were misclassified as indeterminate. For the complete model, these misclassification rates decreased to 2.2% and 2.5%, respectively, while the accuracy for the determinate class remained above 99%. These results indicated that the complete model substantially alleviated confusion between indeterminate and semi-determinate types without compromising determinate-class recognition performance.

**Figure 4:**
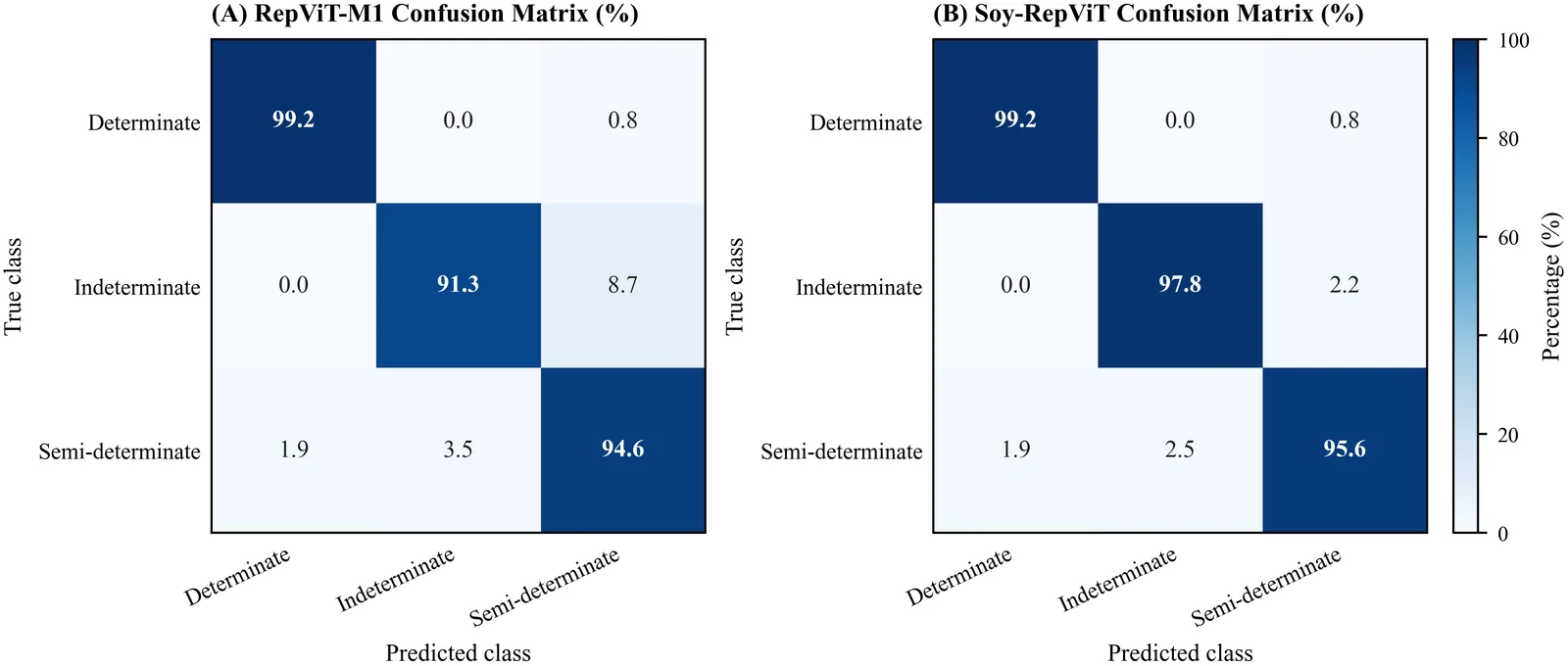
Confusion matrices of RepViT-M1 and Soy-RepViT on the independent test set. **(A)** RepViT-M1. **(B)** Soy-RepViT.

### 3.4 Comparison of attention mechanisms

To evaluate the impact of different channel attention mechanisms on model performance, the plain RepViT-M1 without additional attention modules was used as the baseline. SE (Hu et al., 2018), ECA (Wang et al., 2020), CA (Hou et al., 2021), and the proposed MS-ECA were integrated separately, and comparisons were conducted under the same data partition and training configuration. The results are shown in **Figure 5**. MS-ECA achieved the highest overall accuracy of 94.90%, representing a 0.44 percentage point improvement over the baseline. The most notable gain was observed for the indeterminate class, increasing from 91.30% to 94.05%. SE, ECA, and CA attained overall accuracies of 93.26%, 92.93%, and 92.72%, respectively. These results indicated that MS-ECA more effectively enhanced critical channel responses related to growth habit discrimination, particularly in fine-grained separation between indeterminate and semi-determinate types.

**Figure 5:**
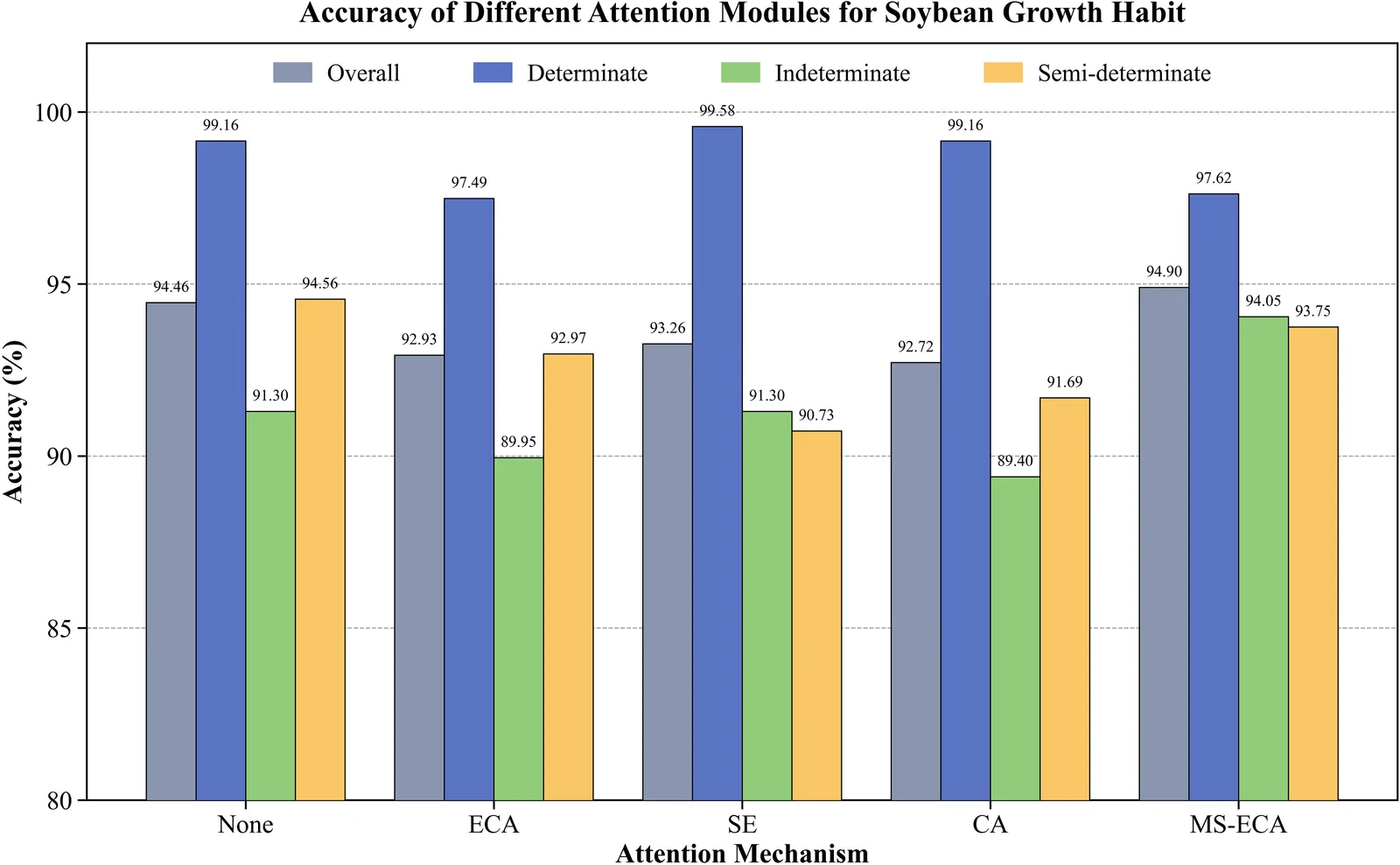
Performance comparison of different attention mechanisms.

### 3.5 Grad-CAM visualization

To further examine how different attention mechanisms affect the regions attended by the model, Grad-CAM was applied to visualize typical samples (Selvaraju et al., 2017), as shown in **Figure 6**. The results indicated that MS-ECA yielded more concentrated high-response regions across all three classes: determinate plants primarily focused on pod-dense areas; indeterminate plants attended more to the main stem apex and upper elongating internodes; and semi-determinate plants showed stronger responses to top pods and adjacent transitional regions. In contrast, models without attention modules or with other attention mechanisms exhibited relatively dispersed response regions. These findings suggested that MS-ECA could suppress responses from irrelevant regions to some extent and strengthen the model’s attention to key agronomic structures such as the main stem apex, thereby helping to alleviate fine-grained boundary confusion between indeterminate and semi-determinate types.

**Figure 6:**
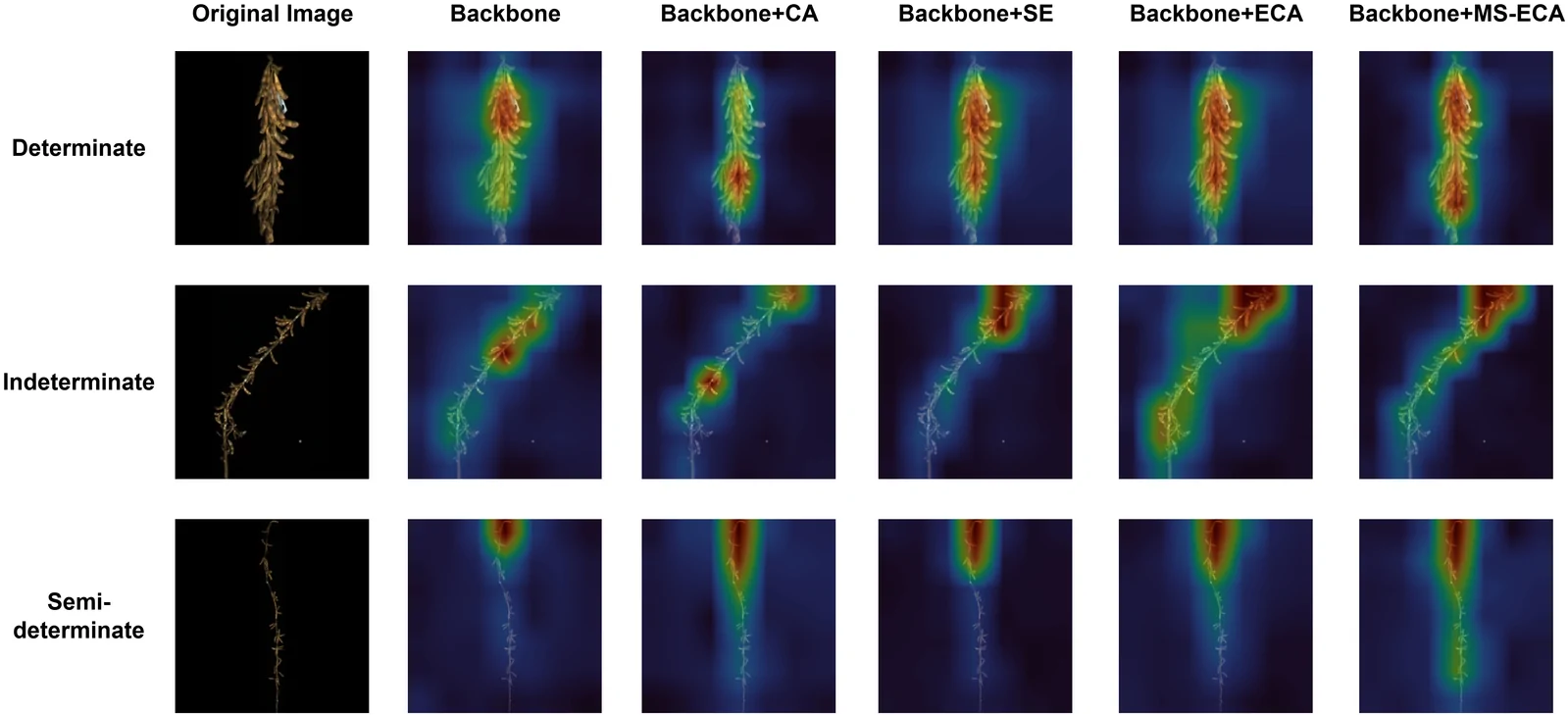
Grad-CAM visualizations for different growth habit categories.

### 3.6 Hyperparameter sensitivity analysis of the FDA module

The FDA module has two key hyperparameters: the hidden-layer dimension ratio *ρ* and the temperature coefficient *τ*. To avoid test-set leakage, different parameter combinations were evaluated using five-fold cross-validation on the training subset, and the FDA hyperparameters were selected based on the mean validation accuracy ± standard deviation. The independent test set was reserved for evaluating the final model configuration. The results are shown in **Figure 7**. With the temperature coefficient τ fixed at 1.0, the mean validation accuracy first increased and then decreased as ρ increased. The highest mean validation accuracy of 94.98±1.20% was achieved at *ρ* = 0.25, whereas accuracies of 94.17±1.13% and 93.56%±1.33% were obtained at *ρ* = 0.125 and 0.5, respectively. These results suggested that an excessively small *ρ* led to overly strong compression of the hidden-layer dimension, which may have caused loss of fine-grained discriminative information. Conversely, an excessively large *ρ* increased feature redundancy and may have introduced noise unrelated to boundary discrimination, thereby weakening the ability of FDA to model intra-class differences within non-determinate types.

**Figure 7:**
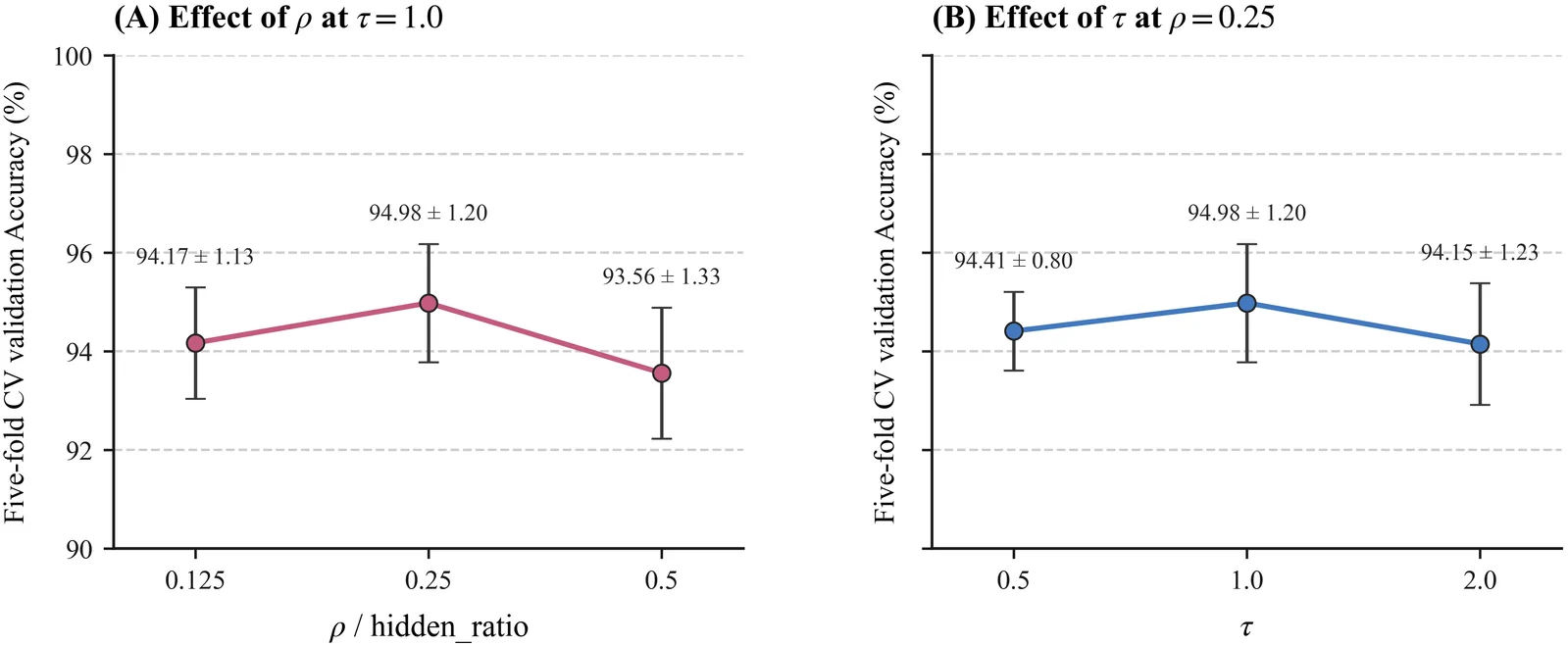
Sensitivity analysis of FDA hyperparameters. **(A)** Hidden-layer dimension ratio *ρ*. **(B)** Temperature coefficient *τ*.

With the hidden-layer dimension ratio fixed at *ρ* = 0.25, the mean validation accuracy also exhibited a trend of first increasing and then decreasing as *τ* increased. The highest mean validation accuracy of 94.98±1.20% was achieved at *τ* = 1.0, whereas the mean validation accuracies of 94.41%±0.80% and 94.15%±1.23% were obtained at *τ* = 0.5 and 2.0, respectively. These results suggested that a smaller *τ* amplified inter-prototype differences, encouraging the model to focus more on local boundary features. Conversely, an excessively large *τ* attenuated inter-class response differences, leading to reduced discriminative sensitivity for boundary samples.

Based on the five-fold cross-validation results, *ρ* = 0.25 and *τ* = 1.0 were selected as the final FDA configuration.

### 3.7 Android application deployment

To assess the practical applicability of the proposed model and its suitability for edge deployment, an Android application was developed for on-device soybean growth habit recognition. After the model configuration was finalized using the training and validation data, the finalized model was converted to the ONNX format and deployed locally on a realme GT Master Explorer Edition smartphone (RMX3366) equipped with a Qualcomm Snapdragon 870 processor and Android 12. The application supports both single-image and batch recognition. For each image, it provides the predicted class, class-wise probabilities, inference latency, and FPS, with the probability distribution displayed as horizontal bars. Recognition results are stored locally on the device, and the prediction history can be viewed and exported as a CSV file. The application operates without a network connection. For mobile inference benchmarking, the batch size was set to 1. The first inference was used as a warm-up and excluded from timing, after which 23 additional images were evaluated. The mean inference latency was 37.50 ms per image, corresponding to an inference speed of 26.67 FPS. These results demonstrate the feasibility of deploying the proposed model for local inference on a consumer mobile device. The application interface is shown in **Figure 8**.

**Figure 8:**
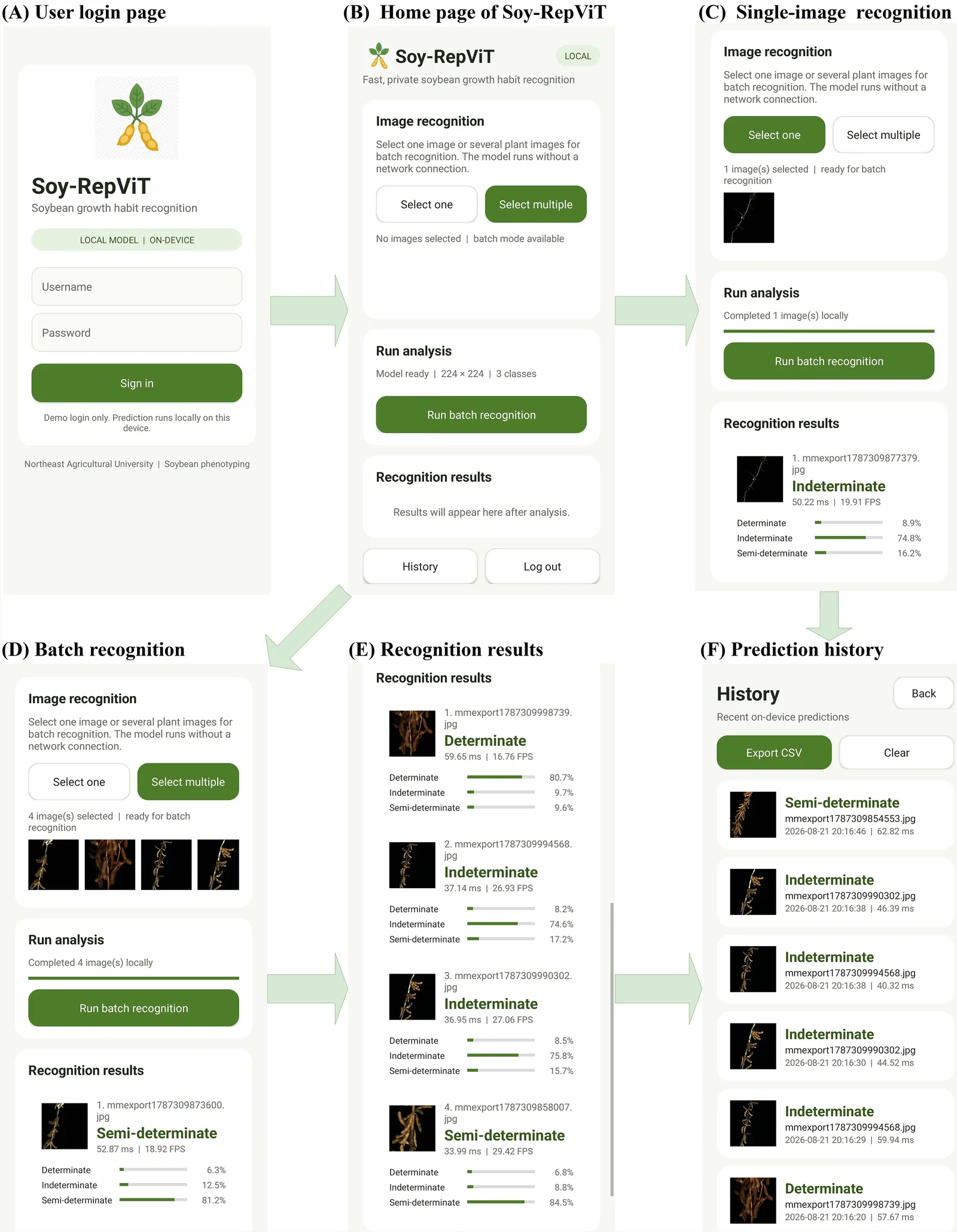
Android application interface. **(A)** User login page. **(B)** Home page of Soy-RepViT. **(C)** Single-image recognition. **(D)** Batch recognition. **(E)** Recognition results. **(F)** Prediction history.

## 4 Discussion

### 4.1 Network construction for fine-grained soybean growth habit recognition

The key to the performance improvement of the proposed method lies not only in the backbone network itself, but also in the recognition pathway tailored to task-specific challenges, consisting of feature enhancement, fine-grained discrimination, and hierarchical discrimination. For determinate samples, the terminal growth characteristics at the main stem apex are relatively distinct, and the plain RepViT-M1 baseline can already discriminate them with reasonable stability. In contrast, indeterminate and semi-determinate types exhibit continuous transitions in main stem apical extension, internode length, and pod distribution patterns, causing a standard flat three-class classifier to learn overlapping decision boundaries. Therefore, the primary challenge of this task is not general three-class classification capability, but rather how to further amplify fine-grained differences within non-determinate types.

To address this issue, this study refined the model structure in three ways: feature enhancement, fine-grained discrimination, and hierarchical discrimination. Specifically, MS-ECA enhances channel responses related to growth habit discrimination based on the high-level features extracted by RepViT-M1, providing a more discriminative feature basis for subsequent fine-grained separation. FDA then performs conditional recalibration of non-determinate features on the enhanced feature representation under the guidance of class prototypes, further highlighting subtle differences between indeterminate and semi-determinate types and reducing confusion between them. Finally, the hierarchical classification head introduces the agronomic prior of determinate versus non-determinate growth habits and decomposes the final decision into coarse-grained and fine-grained stages, making the decision process more consistent with agronomic discrimination logic. In other words, the model first strengthens task-relevant channel information, then improves discrimination between similar non-determinate types, and finally completes hierarchical classification. This progressive design is also an important reason why the complete model reduces confusion between indeterminate and semi-determinate types while maintaining lightweight deployment.

### 4.2 Misclassified sample analysis and interpretability

The confusion matrix indicates that the remaining misclassifications are primarily concentrated between indeterminate and semi-determinate types, whereas recognition of determinate samples remains relatively stable. Combined with analysis of test samples, such errors occur more frequently in boundary samples with atypical phenotypic characteristics. Semi-determinate types inherently exhibit transitional traits, and some samples closely resemble indeterminate types in main stem apical extension, internode length, and pod distribution patterns. Meanwhile, mutual occlusion among pods, overlapping branches, and variations in shooting angle within a single image may prevent the main stem apex region from being fully captured, thereby weakening key discriminative cues. Therefore, these misclassifications do not arise solely from insufficient model representational capacity, but also reflect inherent ambiguity in class boundaries.

Grad-CAM visualization indicates that different attention mechanisms exert a noticeable influence on the regions attended by the model. Compared with models without attention modules or with other channel attention variants, MS-ECA produces more concentrated responses in the main stem apex and adjacent internode regions for indeterminate and semi-determinate samples, while reducing responses in lower pod areas and other plant regions. This suggests that multi-scale channel attention helps shift the discriminative focus toward key agronomic regions more closely associated with growth habit discrimination. Notably, performance changes across classes were not uniform after introducing different attention mechanisms. MS-ECA yielded the most pronounced improvement for the indeterminate class, whereas accuracies for the determinate and semi-determinate classes showed slight fluctuations. This indicates that the model does not simply strengthen features shared across all classes, but allocates more representational capacity to the fine-grained boundary regions between indeterminate and semi-determinate types. Because the terminal inflorescence characteristics of determinate plants are relatively distinct, their classification accuracy was already near saturation in the baseline model. Consequently, the model’s attention focus shifts more toward fine-grained discrimination of complex boundary classes. However, for samples with atypical apical features or incomplete image information, attention shift may still occur even with attention mechanisms, which further supports the necessity of hierarchical decoupling and conditional feature recalibration for addressing fine-grained confusion.

### 4.3 Breeding application value and lightweight deployment

The practical value of the proposed model lies primarily in its ability to rapidly screen large batches of plant images under standardized conditions. For breeding materials, manual plant-by-plant assessment of growth habit is not only time-consuming, but also susceptible to experimenter bias, particularly for boundary samples. The model achieved a recognition accuracy of 97.45% on the independent test set, showing strong agreement with manual annotations. It can efficiently and objectively perform soybean growth habit classification, providing a reliable automated classification tool for soybean phenotypic research.

Meanwhile, the application potential of a model depends not only on recognition accuracy, but also on deployment cost and response speed. Compared with models requiring higher parameter counts and computational costs, the proposed method maintains a relatively high accuracy while preserving a compact parameter size and low FLOPs, together with real-time inference capability. This indicates that the model is suitable not only for offline research analysis, but can also be more readily integrated into standardized imaging platforms or portable recognition devices. For agricultural scenarios that require a balance among accuracy, speed, and hardware resource constraints, this lightweight advantage has clear practical significance and provides a foundation for future integration with automated acquisition, data management, and on-site assisted interpretation workflows (Rui et al., 2024; Das Choudhury et al., 2019).

### 4.4 Limitations and future perspectives

Although the proposed method achieved promising results under standardized acquisition conditions, several limitations remain. First, the current dataset was primarily collected using a standardized imaging platform with relatively stable background, illumination, and shooting distance. Therefore, the present results demonstrate the effectiveness of the model under controlled conditions, whereas its generalization to field conditions with natural lighting, complex backgrounds, and more severe occlusion requires further validation. Second, indeterminate and semi-determinate types inherently exhibit biologically ambiguous boundaries. Relying on a single image for classification may provide insufficient characterization of soybean growth habit traits.

Future work can be pursued in two directions. First, soybean plant images can be collected across regions, years, and cultivars, and model robustness can be evaluated under more complex scenarios to enhance practical applicability (Khoee et al., 2024). Second, multi-view images, temporal phenotypic information, and techniques such as model compression and quantization can be integrated to further improve discriminative stability for boundary samples and reduce deployment costs, thereby supporting broader adoption of the model in real-world breeding scenarios (Dantas et al., 2024).

## 5 Conclusion

This study addresses the requirements for classification accuracy and lightweight deployment in fine-grained soybean growth habit classification by developing a lightweight recognition framework. The framework uses RepViT-M1 as the feature extraction backbone and integrates MS-ECA, FDA, and a hierarchical classification head, thereby improving the discrimination of similar growth habit types while maintaining low computational complexity. After model selection using five-fold cross-validation, the complete model achieved an accuracy of 97.45% on the independent test set, representing an improvement of approximately 3 percentage points over the RepViT-M1 baseline. The model maintained only 4.94 M parameters, 0.82 GFLOPs, and an inference speed of 108.75 FPS. An Android application based on the converted ONNX model was deployed locally on a smartphone to further validate the practical feasibility of the proposed method. By combining lightweight feature extraction, conditional feature recalibration for boundary classes, and hierarchical classification, the proposed method effectively alleviates fine-grained confusion between indeterminate and semi-determinate types. It provides a feasible technical solution for high-throughput, automated identification of soybean growth habit while maintaining high recognition accuracy and effectively controlling deployment costs and computational overhead. The method can directly support germplasm evaluation, breeding selection, and large-scale phenotypic data acquisition, offering practical value for advancing intelligent breeding.

## Data Availability

The datasets presented in this study can be found in online repositories. The names of the repository/repositories and accession number(s) can be found below: The soybean plant image dataset used in this study is publicly available on Kaggle at https://www.kaggle.com/datasets/liuqiernb/soybean-plant-images under the CC BY-NC-SA 4.0 license.
