# Supplementary material for: Soy-RepViT: a lightweight deep learning framework for fine-grained recognition of soybean growth habits
Source: Front Plant Sci. 2026 Sep 30;17:1954156. doi: 10.3389/fpls.2026.1954156 (PMC13661940; doi:10.3389/fpls.2026.1954156)
Supplement: Supplementary file 1 [file Table1.docx]

**Supplementary Material**

**Soy-RepViT: A Lightweight Deep Learning Framework for Fine-Grained Recognition of Soybean Growth Habits**

Dongbo Liu¹, Daohan Cui³˒⁴, Hang Liu^3,4^, Tianyuan Song², Zhanguo Zhang³˒⁴, Qingshan Chen³˒⁴, Fanchuang Li³˒⁴*, Rongsheng Zhu³˒⁴*

¹ College of Intelligence Science and Engineering, Northeast Agricultural University, Harbin, Heilongjiang, China

² School of Resources and Environment, Northeast Agricultural University, Harbin, Heilongjiang, China

³ College of Agriculture, Northeast Agricultural University, Harbin, Heilongjiang, China

⁴ State Key Laboratory of Smart Farm Technologies and Systems, Harbin, China

** Corresponding authors*

This supplementary material provides the five-fold cross-validation results for the ablation experiments and the robustness results obtained with multiple random seeds.

**Supplementary Table S2. Five-fold cross-validation results of the ablation experiments**

| **Configuration** | **Fold 1** | **Fold 2** | **Fold 3** | **Fold 4** | **Fold 5** | **Mean ± SD** |
| --- | --- | --- | --- | --- | --- | --- |
| Baseline | 93.96 | 91.76 | 93.82 | 93.88 | 91.88 | 93.06 ± 1.01 |
| MS-ECA | 94.44 | 91.65 | 93.33 | 95.10 | 93.70 | 93.64 ± 1.17 |
| FDA | 94.32 | 91.88 | 94.18 | 95.43 | 93.94 | 93.95 ± 1.16 |
| Hierarchical head | 95.77 | 91.88 | 94.18 | 95.55 | 93.45 | 94.17 ± 1.43 |
| Hierarchical head + FDA | 95.29 | 91.53 | 95.03 | 94.21 | 94.42 | 94.10 ± 1.34 |
| MS-ECA + hierarchical head | 94.93 | 92.81 | 93.33 | 95.88 | 95.27 | 94.44 ± 1.17 |
| MS-ECA + FDA | 93.03 | 93.65 | 94.59 | 95.42 | 95.21 | 94.38 ± 0.91 |
| All three components | 93.41 | 94.27 | 95.82 | 94.89 | 96.51 | 94.98 ± 1.20 |

**Abbreviations:** MS-ECA, multi-scale efficient channel attention; FDA, fine-grained disambiguation adapter; SD, standard deviation.

**Statistical note:** Values in Fold 1-Fold 5 are validation accuracies (%). Mean ± SD denotes the mean and standard deviation across the five validation folds. The table reports the validation results used to assess ablation trends; the corresponding final model evaluations were conducted on the independent test set after the model configuration was fixed.

**Supplementary Table S3. Results of RepViT-M1 and Soy-RepViT across multiple random seeds**

| **Random seed** | **RepViT-M1 baseline Acc (%)** | **Soy-RepViT Acc (%)** |
| --- | --- | --- |
| 6666 | 94.46 | 97.45 |
| 0 | 94.40 | 97.50 |
| 1 | 92.95 | 96.05 |
| 27 | 93.68 | 96.17 |
| 42 | 94.19 | 96.65 |
| 123 | 94.75 | 96.94 |
| 2026 | 95.18 | 97.47 |
| **Mean ± SD** | **94.23 ± 0.73** | **96.89 ± 0.62** |

**Statistical note:** Values are independent-test-set accuracies (%). The reported mean ± SD was calculated across the seven random seeds shown in the table. The primary experiment used seed 6666; the additional seeds were used to assess the robustness of the baseline and complete model to random initialization and data-splitting variability. For each seed, the same plant-level data-partitioning protocol and evaluation procedure were used.

**Evaluation note:** The independent test set was not used for model selection or hyperparameter tuning. All architectural and hyperparameter decisions were made using the training and validation data before the final evaluations were performed.
